# The Senior Fitness Test for assessing functional fitness in cancer patients and survivors: a cross-sectional study

**DOI:** 10.1038/s41598-026-66221-w

**Published:** 2026-08-07

**Authors:** Frieder Krause, Sofie Nieland, Katharina Graf, Elke Jäger, Lutz Vogt

**Affiliations:** 1 Clinic of Oncology and Hematology, Northwest Hospital, University Cancer Center (UCT), Frankfurt am Main, Germany; 2 Sports Medicine & Exercise Physiology, Goethe-University Frankfurt/Main, Ginnheimer Landstr. 39, 60487 Frankfurt am Main, Germany

**Keywords:** Cancer patients, Functional fitness, Senior Fitness Test (SFT), ECOG performance status, Exercise assessment, Cancer, Health care, Medical research, Oncology

## Abstract

Cancer patients commonly experience declines in quality of life and physical function. The Eastern Cooperative Oncology Group (ECOG) Performance Status Scale is widely used to assess functional status; however, it lacks objective measures of physical fitness. The Senior Fitness Test (SFT) provides a structured and standardized approach to objectively quantify functional abilities. This study aimed to assess functional status in cancer patients using the SFT and to examine its relationship with ECOG performance status. Patients undergoing or having completed anti-cancer treatment were recruited for this study. Multicomponent functional fitness was assessed by a trained exercise scientist using the Senior Fitness Test (SFT), including the 2-min step test, 30-s arm curl, 30-s chair stand, chair sit-and-reach, back scratch and the Timed Up-and-Go Test (TUGT). ECOG performance status was extracted from current medical records or determined by a senior researcher according to official rating guidelines. For each SFT component, individual test results were assigned an age based on normative reference values. Fitness age (FA) was calculated as the mean age across all six SFT measures. A total of *n* = 30 subjects (f = 15, 61.6 ± 12.1y, 16 during & 14 after therapy) participated in this study. Within the present sample, FA differed significantly from chronological age (+ 8.59 years, 95%-CI: 3.65–13.5, *p*<0.001) with no differences between therapy stages. Patients defined as limited in their functional status (ECOG 1&2) showed no significant difference between FA and chronological age compared to patients with ECOG stage 0 (*p*=0.346). The Senior Fitness Test (SFT) is a feasible tool to assess functional status in cancer patients and survivors. Participants exhibited functional deficits equivalent to nearly nine years of age advancement compared with healthy norms, which were not reflected by ECOG performance status and did not differ between patients undergoing treatment and survivors. These findings emphasize the need for objective functional assessments to guide tailored exercise interventions that address long-term limitations and improve quality of life.

## Background

Cancer and its treatment are frequently accompanied by a broad range of adverse effects, including declines in muscle strength, aerobic capacity, flexibility, balance, and the ability to perform activities of daily living, resulting in reduced quality of life and functional independence^1–4^. Large longitudinal studies have demonstrated that cancer survivors—particularly older adults—experience accelerated declines in physical function following diagnosis compared with age-matched controls, with impairments often persisting years beyond treatment completion^5–7^. Objective indicators of physical performance, such as gait speed and grip strength, are consistently lower in older cancer survivors than in individuals without a cancer history^8,9^, and these deficits are associated with increased disability burden, poorer quality of life, and potentially worse clinical outcomes^10,11^.

In routine oncology practice, functional capacity is commonly assessed using the Eastern Cooperative Oncology Group (ECOG) Performance Status Scale. While ECOG is widely applied to guide treatment decisions, it is a single-item, clinician-rated measure that lacks objectivity and granularity, often showing only moderate agreement with standardized physical performance tests and failing to capture specific domains such as strength, mobility, and balance^12,13^. Accordingly, multiple studies have emphasized that objective, performance-based assessments provide complementary and often more sensitive information regarding patients’ functional reserve than ECOG alone^14,15^.

The Senior Fitness Test (SFT) is a validated, standardized functional fitness battery originally developed for older adults that assesses lower and upper body strength, cardiorespiratory endurance, flexibility, agility, balance, and functional mobility. It offers age- and sex-specific normative values that are closely linked to everyday functional independence and enable meaningful comparisons across populations^16–18^. The SFT has been successfully applied in various clinical populations, including breast cancer survivors and mixed oncology cohorts, to objectively quantify functional deficits and to inform individualized exercise interventions^19,20^. This is particularly relevant given the growing evidence that tailored aerobic and resistance exercise improves physical function and selected clinical outcomes in cancer patients and survivors, leading major professional organizations to recommend individualized, progressive exercise training for most people living with and beyond cancer^21,22^.

Against this background, the present study aimed to:

1. evaluate functional fitness in a convenience sample of cancer patients and survivors using the Senior Fitness Test and derive a composite Fitness Age (FA);
2. compare FA with chronological and chronological age; and.
3. examine the relationship between SFT-derived FA, ECOG performance status, and therapy stage.

## Methods

### Study design

This cross-sectional study was conducted in accordance with the Declaration of Helsinki and approved by the local Ethics Committee of the Faculty of Psychology and Sport Science, Goethe University Frankfurt (approval number 2022-72). All participants provided written informed consent prior to enrollment and completed a one-time assessment of the Senior Fitness Test (SFT).

### Participants

Patients during or after anti-cancer treatment were eligible to participate in this trial. Exclusion criteria included an inability to perform exercise testing due to physical limitations or difficulty understanding the test instructions because informed consent procedures and participant information were provided in German only. Recruitment took place between 01/2023 and 05/2023 at single clinical side (a hospital in Frankfurt/Germany), during a period in which institutional COVID-19 infection control measures were still in place. These measures intermittently included restricted access to sensitive hospital areas, mandatory mask-wearing, and routine SARS-CoV-2 testing. Participants were grouped according to current treatment status (during therapy vs.after therapy). Due to clinical heterogeneity in care settings regarding tumor entities, disease stages, and treatment modalities, and the resulting variability in treatment-related effects, no stratified or adjusted analyses were performed. The analysis was therefore exploratory in nature.

### Outcomes

#### ECOG status

The ECOG status is commonly used to determine functional status in cancer patients a six-stage rating scale between normal (ECOG 0) and dead (ECOG 5)^13^. In our sample, ECOG status was extracted from current medical records. When no current medical records were available, ECOG status was determined according to standardized rating instructions^13^ by a senior researcher with fifteen years of experience. The maximum time interval between ECOG-status assessment and functional testing was 10 days. For analysis, ECOG was stratified as fully active (ECOG status = 0) or limited (ECOG status > 0).

#### Senior-Fitness-Test (SFT)

The Senior-Fitness-Test was developed as an assessment tool of functional fitness for older adults in the late 1990s^18,23^. It consists of six independent items to asses multi component functional fitness (for a detailed description see Table 1) and has been shown to be both valid and reliable (ICC between 0.80 and 0.97 for the six items)^18^. It has been used to assess functional fitness in a variety of clinical populations, including patients with brain injury^24^, Parkinson’s disease^25^, cognitive impairments^26^ or breast cancer^19^. After standardized instructions, all participants performed all six items of the SFT in the same order.

**Table 1:** Senior Fitness Test (SFT) components: six independent items.

Item	Description	Outcome
Chair Stand	Sitting on a chair with the arms folded across the chest, the patient stands up and sits down for a total of 30 s	Number of repetitions [n], representing lower body strength
Arm Curl	Using a 2.3 kg (female) od 3.6 kg (male) dumbbell, patient executes arm curls sitting on a chair for 30 s	Number of repetitions [n], representing upper Body strength
Chair Sit-and-Reach	Sitting at the edge of the chair with one leg extended, the patient tries to reach toward the toes with both hands	Distance [cm] between fingertip of middle finger and toes, representing lower body flexibility
Back Scratch	In a standing position, the patient reaches over the shoulder with one hand and up the middle of the back with the opposite hand	Distance [cm] between fingertips of both middle fingers, representing upper body flexibility
Timed Up-and-Go	From a seated position, the patients stands up, walks 2.4 m, turns around a cone, walks back and returns to seated position	Time [s], representing overall agility and balance
2-Minute-Step	In a standing position, the patient alternately lifts the knees to the minimum height (halfway between patella and spina iliaca anterior superior) for two minutes	Number of times [n], that the right knee reached the minimum height, representing aerobic endurance

#### Fitness-Age (FA)

For each item, Fitness Age (FA) was calculated using sex-specific normative values^23^ and defined as the midpoint of the age band corresponding to the 50th percentile of the individual test result. For values above or below the normative range, the youngest or oldest age of the adjacent category, respectively, was assigned as the FA.

The mean of the six items was calculated, resulting in a global FA for each participant.

Difference between FA and chronological age was calculated to quantify impairments in functional fitness, defined as a Fitness Gap (FG).

### Statistical methods

Following verification of statistical assumptions, differences between chronological age and Fitness Age (FA) were evaluated using paired-samples *t*-tests. Comparisons of fitness gaps across therapy stages (during vs. post-treatment) and ECOG performance status categories (fully active vs. limited) were performed using independent-samples *t*-tests. Effect sizes were calculated according to Cohen’s *d*. Statistical analyses were conducted using Jamovi (version 2.4.1), with a significance threshold set at α = 0.05.

## Results

### Participants

During the study period, a total of 30 participants provided written informed consent and were enrolled in the study. Given an average quarterly patient volume of approximately 700–800 patients in the Department of Oncology and Hematology, and an above-average proportion of patients with limited German language proficiency in the local population, the achieved recruitment rate is consistent with the frequently reported clinical trial participation rate of approximately 10–15% in specialized oncological care settings, across varying oncological entities. Characteristics of the study sample and diagnoses are displayed in Table 2.

**Table 2:** Demographic and diagnosis-related characteristics.

Sample Characteristics	
Age [y] (mean ± SD)	61.6 ± 12.1
Female [n]	15
Male [n]	15
Height [cm] (mean ± SD)	173 ± 10
Weight [kg] (mean ± SD)	78.8 ± 23.0
BMI [kg/m^2^] (mean ± SD)	25.9 ± 5.8
During therapy [n]	16
After therapy [n]	14
Time since end of treatment (range [months])	5–26
ECOG 0 [n]	23
ECOG > 0 [n]	7
UICC stage I [n] (during therapy [n])	2 (0)
UICC stage II [n] (during therapy [n])	9 (2)
UICC stage III [n] (during therapy [n])	8 (3)
UICC stage IV [n] (during therapy [n])	11 (11)
Diagnose Sites [n]	
Lung	6
Breast	6
Colon	5
Pancreas	4
Stomach/esophagus	2
Rectum	2
Lymphoma	2
Prostate	2
Bone marrow	1
Treatment type of patients during therapy [n]	
Chemotherapy	7
Immunotherapy	4
Combined Chemo-Immunotherapy	4
Anti-Hormonal therapy	1

### Senior Fitness Test

**Fig. 1:**
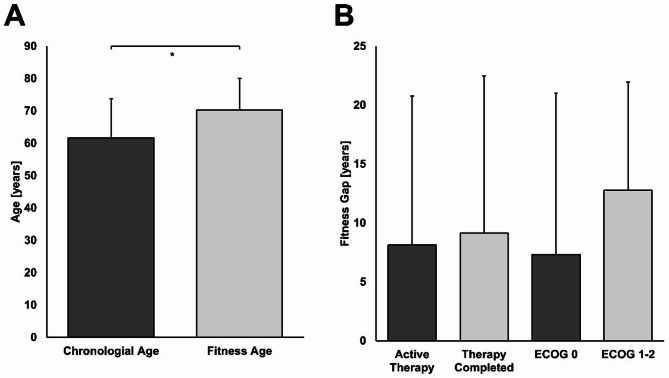
Fitness Age and fitness gaps in the study population. (**A**) Comparison of chronological age with mean Fitness Age (FA). (**B**) Fitness gaps (FG) stratified by therapy stage and ECOG performance status.

All participants successfully completed all Senior Fitness Test (SFT) components, with no adverse events reported (see Table 3). The mean Fitness Age (FA) of the sample was 69.5 ± 9.8 years, which was significantly higher than chronological age (*p* < 0.001, Cohen’s d = 0.65; Fig. 1A). The mean fitness gap (FG) was 8.59 years (95% CI: 3.65–13.5).

**Table 3:** Results of individual items of the SFT and calculated fitness-age and -gap.

Item	SFT score (mean ± SD)	Fitness Age [years] (mean ± SD)	Fitness Gap [years] (mean ± SD)
Chair Stand	15.0 ± 5.1	71.5 ± 15.4	9.9 ± 20.7
Arm Curl	16.9 ± 4.9	71.0 ± 14.7	9.4 ± 19.6
Chair Sit-and-Reach	4.5 ± 10.6	66.7 ± 18.9	5.1 ± 23.8
Back Scratch	−7.6 ± 14.0	69.1 ± 19.9	7.4 ± 19.0
Timed Up-and-Go	5.2 ± 1.4	66.2 ± 13.0	4.6 ± 13.8
2-Minute-Step	81.6 ± 22.4	77.7 ± 16.3	16.1 ± 20.1

### Therapy stages and ECOG status

Within the present sample, no statistically significant differences in Fitness gap (FG) were observed between participants during and after therapy (8.11 ± 13.1 vs. 9.13 ± 13.9; *p* = 0.838, d = 0.08) or across ECOG performance status categories (ECOG 0: 7.31 ± 14 vs. ECOG > 0: 12.8 ± 9.93; *p* = 0.346, d = 0.41; Fig. 1B).

## Discussion

Building on the study’s objective to assess functional fitness using the Senior Fitness Test (SFT) and to compare its results with ECOG performance status, our findings demonstrate that the SFT is a feasible, safe, and sensitive tool for objectively evaluating functional status in cancer patients and survivors. No adverse events occurred during testing, indicating that the SFT can be safely applied both during and after anti-cancer treatment. Test completion required approximately 20–30 min, depending on the need for individual explanation of the six test components, which supports its practicality in clinical or research settings. These findings are consistent with previous studies in oncological populations reporting high feasibility and responsiveness of the SFT^19^.

Beyond feasibility, the SFT identified substantial multi-domain functional impairments when compared with established age- and sex-specific normative values. On average, participants demonstrated a Fitness Gap (FG) of 8.59 years, indicating that their functional performance corresponded to normative values typical of individuals nearly nine years older. This finding provides an integrated quantification of cancer- and treatment-related declines across strength, endurance, flexibility, balance, and mobility. Similar impairments have been reported in previous studies showing that cancer survivors exhibit lower gait speed, reduced grip strength, and poorer overall physical performance than age-matched individuals without cancer^8,27^. These deficits may reflect a combination of treatment-related toxicities, chronic inflammation, deconditioning, and accelerated aging processes, which could compound normal age-related declines in physical function^28^.

Notably, the magnitude of the FG did not differ between patients undergoing active therapy and those in the post-therapy phase, suggesting that cancer-related impairments in functional fitness may persist well beyond treatment completion. This finding aligns with longitudinal evidence demonstrating sustained or progressive declines in functional capacity among cancer survivors compared with cancer-free populations. It underscores the need for long-term, individualized exercise and rehabilitation strategies targeting persistent deficits in physical function and fitness^22^. However, given the single time-point assessment in the present study, interpretations regarding differences between therapy stages are based on normative comparisons and existing literature rather than within-subject temporal data.

Interestingly, SFT-derived FG did not differ significantly between patients classified as fully active (ECOG 0) and those with mild functional limitations (ECOG > 0). Although mean FG values were lower in patients without ECOG-defined limitations, high interindividual variability resulted in non-significant group differences. This finding underscores the possibility that ECOG performance status, as a subjective single-item clinician-rated scale, may not fully capture physiologic reserve or functional domains relevant to daily activities. Prior research has shown only moderate correlations between ECOG scores and objective performance measures, with a substantial proportion of patients rated as ECOG 0–1 still exhibiting impairments in strength, mobility, balance, or ADL/IADL function^12,29^. Our results add to this body of evidence by demonstrating that structured, performance-based assessments such as the SFT can reveal clinically meaningful impairments that are not captured by ECOG alone.

From a clinical perspective, these findings support the integration of brief, validated objective assessments – such as the SFT, gait speed^30^, timed up-and-go^31^, or the 6-minute walk test^32^– into routine oncological care. Such tools may facilitate earlier identification of subclinical functional impairments and enable timely referral to targeted exercise or rehabilitation programs^2^. Furthermore, domain-specific results from the SFT can support the development of tailored exercise programs targeting strength, endurance, balance, or flexibility, in line with international exercise recommendations for people living with and beyond cancer^22^. In addition to these domain-specific outcomes, the composite Fitness Age derived from the SFT subtests may provide a broader summary measure of functional status and serve as an indicative screening index for functional limitations. As a screening approach, it captures multiple physiological systems simultaneously, including strength, endurance, balance, and flexibility. Deficits in any single domain reduce the overall performance profile, allowing early functional limitations to be detected across different areas of physical functioning. This makes it suitable for identifying potential indicators of fall risk, general frailty, and training needs. However, it should be emphasized that this does not represent a validated measure of biological age, but rather an aggregated deviation from age-related normative values across multiple domains of motor performance.

Future research should investigate whether SFT-derived measures, including the proposed Fitness Gap construct, predict clinically relevant outcomes such as treatment tolerance, hospitalization, functional decline, or survival. Longitudinal and randomized controlled trials are also needed to determine whether targeted exercise interventions that reduce these objective impairments translate into improved clinical endpoints and long-term survivorship outcomes.

Several limitations should be acknowledged, including the small sample size, heterogeneous patient population, and lack of a control group, which limit statistical power and generalizability. The cross-sectional study design precludes causal or longitudinal interpretations. Also, feasibility and safety can only be concluded for participants meeting our inclusion criteria, further limiting generalizability. In addition, ECOG performance status was not consistently assessed by the attending physician but by a senior researcher (in cases where no ECOG was available in the medical records), so the possibility of classification inaccuracy cannot be fully excluded. Factors that may affect physical performance and functional outcomes, including certain comorbidities and lifestyle-related factors such as physical activity, nutrition, and smoking status, were not systematically assessed in this study and may have influenced the observed results. It should also be noted that the study population was clinically heterogeneous with respect to tumor entities, disease stages, and treatment modalities. Although this variability may limit the generalizability of the findings, it reflects real-world oncological care settings. In addition, comparisons between participants during and after therapy were performed without adjustment for these factors, as adjustment was not methodologically appropriate given the clinical heterogeneity of the study population. Consequently, potential diagnosis- or treatment-specific effects on functional fitness cannot be excluded and may have been masked by cohort variability. With regard to the overall study cohort, participant enrollment may partly reflect the impact of institutional COVID-19 infection control measures in place during parts of the recruitment period.

Despite these limitations, the assessment proved feasible in routine clinical practice. Following appropriate training and familiarization with the measurement protocol, it could be integrated into the clinical workflow with minimal disruption and without placing a substantial burden on patients or clinical staff. Future studies with larger samples are needed to confirm these findings and further evaluate their generalizability.

Overall, the present findings highlight the added value of objective, multi-domain functional assessment in oncology and support the SFT as a meaningful complement to traditional performance status scales.

## Conclusion

Our results indicate that cancer patients, both during active treatment and after therapy completion, experience clinically relevant losses in functional fitness and physical function compared to normative values of healthy adults that are not adequately captured by ECOG performance status. These findings herewith point to the potential value of complementing subjective performance scales with objective assessments, such as the Senior Fitness Test, to better characterize functional status in cancer patients and survivors. Integrating objective functional measures into clinical care can facilitate the early identification of deficits and inform the development of individually tailored exercise interventions targeting persistent impairments, with the potential to improve long-term functional independence and quality of life in cancer patients and survivors.

## Data Availability

The datasets used and/or analysed during the current study are available from the corresponding author on reasonable request.
